# Choice of Alternative Polyadenylation Sites, Mediated by the RNA-Binding Protein Elavl3, Plays a Role in Differentiation of Inhibitory Neuronal Progenitors

**DOI:** 10.3389/fncel.2018.00518

**Published:** 2019-01-10

**Authors:** Elena Grassi, Roberto Santoro, Alessandro Umbach, Anna Grosso, Salvatore Oliviero, Francesco Neri, Luciano Conti, Ugo Ala, Paolo Provero, Ferdinando DiCunto, Giorgio R. Merlo

**Affiliations:** ^1^Department of Molecular Biotechnology, University of Turin, Turin, Italy; ^2^Department of Neurosciences, University of Turin, Turin, Italy; ^3^Italian Institute for Genomic Medicine, Turin, Italy; ^4^Department of Life Science and System Biology, University of Turin, Turin, Italy; ^5^Centre for Integrative Biology—CIBIO, University of Trento, Povo, Italy

**Keywords:** neuron differentiation, GABAergic, polyadenylation, RNA binding protein, Elavl3

## Abstract

Alternative polyadenylation (APA) is a widespread mechanism involving about half of the expressed genes, resulting in varying lengths of the 3′ untranslated region (3′UTR). Variations in length and sequence of the 3′UTR may underlie changes of post-transcriptional processing, localization, miRNA targeting and stability of mRNAs. During embryonic development a large array of mRNAs exhibit APA, with a prevalence of the longer 3′UTR versions in differentiating cells. Little is known about polyA+ site usage during differentiation of mammalian neural progenitors. Here we exploit a model of adherent neural stem (ANS) cells, which homogeneously and efficiently differentiate into GABAergic neurons. RNAseq data shows a global trend towards lengthening of the 3′UTRs during differentiation. Enriched expression of the longer 3′UTR variants of *Pes1* and *Gng2* was detected in the mouse brain in areas of cortical and subcortical neuronal differentiation, respectively, by two-probes fluorescent *in situ* hybridization (FISH). Among the coding genes upregulated during differentiation of ANS cells we found *Elavl3*, a neural-specific RNA-binding protein homologous to *Drosophila* Elav. In the insect, Elav regulates polyA+ site choice while interacting with paused Pol-II promoters. We tested the role of Elavl3 in ANS cells, by silencing *Elavl3* and observed consistent changes in 3′UTR length and delayed neuronal differentiation. These results indicate that choice of the polyA+ site and lengthening of 3′UTRs is a possible additional mechanism of posttranscriptional RNA modification involved in neuronal differentiation.

## Introduction

In neural progenitor cells, exit from cell cycle and initiation of neuronal differentiation is a complex process, whose fine regional regulation assures the timely generation of distinct neuronal and non-neuronal types composing the final functional networks. Big progress has been made toward the understanding of molecular mechanisms directing and controlling cell cycle exit, commitment and early differentiation (Hardwick et al., 2015).

Neuronal differentiation has been examined at the level of dynamics of transcriptome repertoire. Extensive maps of gene expressions and gene/protein interactions have been derived from omic data, relative to region-specific normal neuronal differentiation (Lein et al., 2007; Hawrylycz et al., 2012; Miller et al., 2014, 2017; Bakken et al., 2016) as well as to disease conditions (Yano et al., 2015). Components of the cell cycle control machinery are heavily implicated, as expected. However, the transcriptome and its dynamic changes have turned out to be far more complex than previously thought. First of all, evidence indicates the existence of multiple classes of RNAs such as microRNAs (miRs), long non-coding RNAs (lncRNAs) and circular RNAs (circRNAs; Ji J. et al., 2009; DeWitt et al., 2016; Suiko et al., 2016; Rajman and Schratt, 2017; Lennox et al., 2018). Second, although the control of gene transcription at the promoter level certainly represents a key regulatory step, the complexity of the transcriptome is largely increased by additional layers of cotranscriptional and posttranscriptional regulations, including miRs-mediated silencing, competitive-endogenous RNA (ceRNA) networks, alternative splicing, non-sense mediated RNA decay (NMD) and alternative polyadenylation (APA). Furthermore the various classes of regulatory molecules intersect via RNA:RNA and RNA:protein cross-regulations via complex, only partially known, mechanisms (Dai et al., 2015; Chen and Schuman, 2016; Hanan et al., 2017; Lara-Pezzi et al., 2017; Wanke et al., 2018).

The control of neuronal commitment and early steps of differentiation utilizes all of these emerging RNA classes and regulatory mechanisms (Lukovic et al., 2014; Stappert et al., 2015; Rajman and Schratt, 2017). miRs are critically involved in conferring neural cell identities during neural induction, neuronal differentiation and subtype specification (Stappert et al., 2015; Rajman and Schratt, 2017). miR-124, is probably the most well-documented example of a miR that controls nerve cell fate determination (Makeyev et al., 2007; Åkerblom and Jakobsson, 2014). let-7 and miR-9 have also been shown to promote the differentiation of neural stem and neural progenitor cells into specific neural cell types, while miR-134, -25 and -137 induce their proliferation (Meza-Sosa et al., 2014; Roese-Koerner et al., 2017). Recent studies have uncovered that endogenous RNAs competing for binding to miRs (ceRNAs) control a larger number of miR target transcripts (Gardiner et al., 2015). The role of ceRNA networks in neuronal differentiation is still unexplored.

RNA splicing plays a critical role in the programming of neuronal differentiation and its disruption may underlie neurodevelopmental and neuropsychiatric disorders (Lara-Pezzi et al., 2017). RBFOX1 is a neuron-specific RNA-binding protein (RBP) that coordinated splicing events relevant for neuronal development as well as clinically important transcriptional programs (Fogel et al., 2012). Splicing factors can rapidly increase the production of mRNAs encoding proteins important for synaptogenesis (Yap et al., 2012; Zheng et al., 2012). A functional interaction between miR-based regulations and alternative pre-mRNA splicing has been recognized. For instance miR-124 promotes neural differentiation at least in part by regulating an intricate network of brain-specific alternative splicing events (Makeyev et al., 2007; Lennox et al., 2018).

Neurogenesis and neuronal wiring have been shown to involve nonsense-mediated decay (NMD; Lara-Pezzi et al., 2017). For instance, the NMD control protein RBM8a is involved in the regulation of proliferation and differentiation of neural progenitors, and autism risk genes are highly represented among putative downstream targets identified by RNAseq profiling (Zou et al., 2015).

Likewise, mutations in the NMD core factor gene UPF3B are associated with neurodevelopmental disorders including X-linked intellectual disability, autism, childhood onset schizophrenia and attention deficit hyperactivity disorder. Expression of missense mutant UPF3B disturbs neuronal differentiation and reduces neurite complexity (Alrahbeni et al., 2015). Loss of UPF3B in neural progenitor cells causes expansion of cell numbers at the expense of their differentiation (Jolly et al., 2013). The control of NMD has been linked to the expression and function of specific miRs, such as miR-128, suggesting the existence of RNA circuits linking the miR and NMD pathways to expression of cell type-specific transcripts during neuronal differentiation (Bruno et al., 2011; Karam and Wilkinson, 2012).

Finally, circRNA levels are dynamically modulated in neurons, both during differentiation and following bursts of electrical activity, and accumulate with age. Many circRNAs are enriched in synapses. Currently available data suggest that circRNAs play important roles in synaptic plasticity and neuronal function and thus represent a novel RNA-based regulation, still poorly studied (Rybak-Wolf et al., 2015; Chen and Schuman, 2016; Hanan et al., 2017).

One emerging mechanism that generates further molecular diversity in the RNA repertoire is the alternative choice of polyadenylation sites at the 3′ end of transcribed units, known as APA. The extent of the APA in specific biological processes has been highlighted and reviewed by several reports (Elkon et al., 2013; Akman and Erson-Bensan, 2014; Brumbaugh et al., 2018). In general, in proliferating cells, hundreds of transcripts preferentially terminate at the upstream polyA+ site and lead to shorter 3′ untranslated region (3′UTR) variants (Sandberg et al., 2008; Ji Z. et al., 2009; Mayr and Bartel, 2009). Conversely, differentiating cells preferentially chose a distal polyA+ site with consequent lengthening of 3′UTR. The impact of APA on cellular processes is beginning to be gazed to this day (Elkon et al., 2013; Tian and Manley, 2013; Akman and Erson-Bensan, 2014; Chen et al., 2017). One hypothesis could be that the transcribed sequences that are included or excluded by alternative polyA+ usage may confer differential stability to the transcript, may harbor miRNA target sequence (Blazie et al., 2017), may target mRNA isoforms to specific subcellular locations, or may engage in alternative secondary structures that may influence translation (Berkovits and Mayr, 2015).

During brain development, elongation of the 3′UTR is an extensive phenomenon and has been documented in *Drosophila* (Hilgers et al., 2012), in zebrafish (Ulitsky et al., 2012) and in mammals (Hilgers et al., 2012; Miura et al., 2013). One notable example is *Bdnf* mRNA; its two 3′UTR isoforms each have distinct functions in neurons. The long *Bdnf* isoform is localized to dendrites and translated upon neuronal activity, whereas the short isoform is localized to the cell body and is constitutively translated. Mice that lack the long 3′UTR of *Bdnf* exhibit altered dendritic spine morphology and decreased plasticity of dendritic synapses (An et al., 2008; Lau et al., 2010). Another notable example: the expression of longer 3′UTR of the *Rac1* mRNA (a key small GTPase involved in neuronal maturation) is a gene- and cell type-specific mechanism in the brain (Braz et al., 2017). These authors demonstrated that the longer 3′UTR of the *Rac1* mRNA is required for driving the mRNA to the neurites and for neurite outgrowth of cortical neurons (Braz et al., 2017). Even in neural development, shorter 3′UTR have been associated to the cell proliferation (Sandberg et al., 2008) while the usage of distal polyA+ sites correlates with cell differentiation and organism development (Ji Z. et al., 2009; Shepard et al., 2011; Wang et al., 2013; Tallafuss et al., 2015).

The extent of these phenomena and whether they are regionally-restricted and cell type-dependent are issues that remain to be clarified. APA has been examined in mature neurons, comparing distinct cell types (Braz et al., 2017; Jereb et al., 2018), and during neuronal activation (Flavell et al., 2008). However, a step-wise analysis of APA in early commitment and differentiation of neural progenitor is lacking. In particular, basal-type neural progenitors committed to the inhibitory neuronal fate have not been examined. Here we report transcriptome-wide changes of the length of 3′UTR during differentiation of mouse adherent neural stem (ANS) cells, a model of differentiation of GABAergic inhibitory neurons (Pollard et al., 2006; Paina et al., 2011). We detect a consistent change in the polyA+ site usage in cultured ANS cells, as well as in the developing mouse brain. We also establish a role of Elavl3, showing that Elavl3-mediated control of 3′UTR length contributes to differentiation of inhibitory neuron. These results indicate that lengthening of 3′UTRs is an additional mechanism of posttranscriptional RNA modification involved in neuronal differentiation.

## Materials and Methods

### Growth and Differentiation of ANS Cells

ANS cells were derived from normal mouse embryonic brain, at the age E14.5, characterized and used according to published procedures (Pollard et al., 2006; Onorati et al., 2011; Paina et al., 2011). Briefly, ANS cells were maintained and expanded in a medium (named growth medium) consisting of Euromed-N medium (Euroclone, Celbio) supplemented with 1% N2 (Invitrogen), 20 ng/ml of hrFGF2 (Peprotech) and 20 ng/ml of hrEGF (Peprotech). For differentiation to GABAergic neurons, confluent ANS cells were gently dissociated using Accutase (Sigma) and plated at 1.0 × 10^5^–1.5 × 10^5^ cells/cm^2^ in expansion medium, after 1 day the medium was changed with a medium (named medium D1) consisting in Euromed-N with 0.5% N2, 1% B27 (Invitrogen), 10 ng/ml hrFGF and 2 ng/ml of hrEGF for 3 days. After that, cells were again dissociated with Accutase and seeded at density of 5–7.5 × 10^4^ cells/ml onto laminin-coated (2 mg/ml) dishes and maintained in a medium (named medium B) consisting of a 1:3 mix of DMEM/F12 and Neurobasal media (Invitrogen) containing 0.5% N2 and 1% B27 supplements, FGF-2 (10 ng/mL) and BDNF (20 ng/mL) for 3 days.

### RNA-seq on Differentiating ANS Cells

Cells were collected at time 0 (T0, proliferating conditions), time 1 (T1, reduced proliferation), time 2 (T2, begin differentiation) and time 3 (T3, late differentiation), in biological triplicates, and used to extract total RNA, with Trizol, according to standard procedures. RNA-seq was performed as recently described (Neri et al., 2015). Libraries were generated using TruSeq RNA Sample Prep kit v2, and then sequenced on Illumina platform HiScanSQ. Basecalls was performed using CASAVA version 1.8.

### Analyses of RNA-seq Data

Alignments were performed using TopHat (version 2.0.6, with samtools 0.1.18.0 and Bowtie 0.12.7.0, Trapnell et al., 2012) using genome indexes built with bowtie2-build on chromosomal sequences downloaded from UCSC mm9. We obtained an average percentage of reads mapped of 94.6%. Differentially expressed genes (DEGs) were called using DESeq2 (Anders and Huber, 2010) with the reference gtf for mm9 downloaded from the Illumina iGenomes project and a 0.05 FDR threshold. Functional enrichment analyses were run using genes found up or down-regulated in the various comparisons between differentiated and not differentiated samples and the GO database (Ashburner et al., 2000)—Fisher test *p*-values were corrected to account for the multiple test issue with Bonferroni and only G.O. terms with a corrected *p*-value lower than 0.05 were reported.

The analysis of APA site usage has been performed with the roar Bioconductor package (Grassi et al., 2016). In brief, this package reports for every studied gene a value (roar) representing how the short and long isoforms levels changes across the two compared conditions and a *p*-value reflecting the significance of this imbalance. As an annotation source for APA sites we used PolyaDB2 (Lee et al., 2007) and studied every gene with at least an APA site considering as “long” the isoform with the same end as the canonical transcript reported by the NCBI Refseq track and as “short” the one ending with the most 5′ proximal APA site annotated for that gene in the 3′-UTR (i.e., the one that will result in the shortest isoform possible). When a transcript did not have an APA in the 3′UTR (6.7%) we applied the same rule. At the gene level, for every gene we chose its longest Refseq transcript. With this pipeline we were able to examine the polyadenylation status of a total of 11,945 transcription units.

Transcript variants were classified as shortened or lengthened according to the following criteria: 1. a roar value > 1 (shortening) or < 1 (lengthening); 2. all possible comparisons yield a nominal *p*-value < 0.05; 3. cutoff on the expression levels of the common sequence (present in both the short and long transcript variants) of the gene: FPKM > 1. This limit applies to both conditions. With all possible comparisons we refer to all the pairings between samples obtained at the chosen differentiation times.

### HIT CLIP Data Enrichment Analyses

Elavl binding sites (mm9 coordinates) were obtained from Supplementary Table S2 of Ince-Dunn et al. (2012). We examined the number of overlaps between these binding sites and the shortened and lengthened genes found in the time course of differentiating ANS cells. We examined overlaps on the entire genomic coordinates, instead of on transcripts only, since the mechanisms of Elavl3 activity on regulation of APA are not completely known. We then performed a Fisher test to examine whether the lengthened (or shortened) genes are enriched in Elavl3 targets when compared with the whole list of analyzed genes.

The biological significance of this test is further supported observing that enrichment *p*-value for the lengthened genes become more significant (1.77e-5 vs. 0.0011) when we restricted our analysis to the robust clusters of Elavl binding as defined in Ince-Dunn et al. (2012) using FDR < 0.01 or BC ≥ 5). The enrichments were never significant for the shortened genes.

### siRNA-Mediated Downmodulation of Elavl3 in ANS Cells

For silencing of *Elavl3* mRNA, we used Accell SMART pool siRNA oligonucleotides (Dharmacon), known to be cell-permeable without the need of transfection, and to be more stable as compared to other systems. The pool of siRNA was designed and synthesized by Dharmacon, on the target sequences reported in Supplementary Table S1A. siRNA oligonucleotide were used at final concentration of 1 μM. The siRNAs were added to ANS cells during the last day in expansion medium and in the D1 medium for the remaining 3 days. For Western blot analyses, total protein extracts from proliferating ANS cells were prepared with a lysis buffer 2% sodium dodecyl sulfate, 30% glycerol, 300 mM *β*-mercaptoethanol, 100 mMTris-HCl pH 6.8 followed by polyacrylamide gel electrophoresis and transfer to PVDF, according to standard protocols. Anti-Elalv3 antibody used was from Proteintech Europe (55047-1-AP), used 1:1,000, revealed with a conjugated secondary anti-rabbit (Santa Cruz). Images were quantified by digital densitometric analysis using Chemidoc Touch Imaging system (BIO-RAD).

### Real-Time qPCR on Differentiating ANS Cells

ANS cells were expanded, plated in 6-wells plastic clusters and differentiated *in vitro* as indicated above. The sample named “Time 0” consisted in proliferating ANS cells maintained in growth medium. The sample named “Time 3” consisted in ANS cells differentiated for 4 days in medium B. Total RNA was collected at two time-points (time 0 and time 4) from the initial differentiation step, by removing the culture medium followed by addition of 0.5 ml of the reagent Trizol (Invitrogen) and scraping. The sample were collected in Trizol and used to extract total RNA according to the instructions.

To determine the relative abundance of specific mRNAs and for validation of the RNAseq data, Real-Time qPCR was used on independently collected samples. Two-hundred and fifty nanogram of total RNA was reverse-transcribed at 42°C for 50 min in the presence of 500 ng/μl random hexamers, 10 mM of each dNTPs, RNasin and Improm Reverse Transcriptase (Promega). Relative cDNA abundance was determined using the AB7900 System and the Platinum SYBR GREEN qPCR Super Mix (Life Technology). Specific cDNAs were amplified using primers and probes designed according the Universal Probe Library system (UPS, Roche). Experiments were repeated at least twice on independent samples, every point was done in triplicate, results were normalized to the level of *GAPDH* mRNAs. Data analysis was performed with ABI software, version 2.1 (Applied Biosystems). Primer sequences are provided (Supplementary Table S1B).

To determine the relative abundance of the long/short 3′UTR forms of selected mRNAs, a RealTime qPCR-based strategy was used, in which one primer-pair was specifically designed to amplify the long form, while a separate primer-pair was used to amplify both the “long” and the “short” forms, e.g., the total amount of the mRNA. For the calculations, data were normalized with an internal control (GAPDH) and the time 0/time 4 (ANS cells/differentiating cells) ratio was calculated.

### Two-Probes Fluorescent *in situ* Hybridization

A dual-probe fluorescent *in situ* hybridization (FISH) method was used to examine the expression and localization of the common- and long variant forms of *Pes1* and *Gng2* transcripts. In both cases, one probe was designed to anneal to a sequence in the 3′UTR of the long variant, not present in the short variant, and therefore able to detect only the long variant (i.e., distal polyA+ site usage). The other probe was designed to recognize both the long and the short variant transcripts together (proximal + distal polyA site usage). The short variant (i.e., use of proximal polyA+ site) cannot be unequivocally detected with this method. For the *Pes1* mRNA the common probe detected a sequence that anneals with exons XIII to XV, while for the *Gng2* mRNA the common probe detected a sequence that anneals with exons III to VI. The probe sequences were generated by PCR amplification (primer sequences and amplicon information are provided in Supplementary Table S1C) from mouse total cDNA, followed by standard plasmid cloning and sequence verification. For the generation of the labeled RNA probes, the plasmids were linearized with SpeI, purified and used for *in vitro* transcription with the DIG RNA Labeling kit (SP6/T7; Roche, 11175025910), in the presence of fluorescein-UTP (incorporated into the 3′UTR *Pes1* probe) or digoxigenin-UTP (incorporated into the common *Pes1* probe). The yield and integrity of labeled RNA was confirmed by gel electrophoresis, the probes were purified by spin chromatography.

Embryonic E14.5 brains were snap-frozen in liquid N2 and cryosectioned at 18 μm following a coronal orientation. The FISH followed published procedures (Grosso et al., 2015). Briefly, sections were incubated with the DIG-labeled riboprobes followed by incubation with anti-DIG–POD (1:500, Roche, 11207733910) and development with cyanine-3 substrate kit (1:200, NEL744001KT, PerkinElmer). Slides were treated with 2% H_2_O_2_ to quench residual POD activity, and hybridized with the fluorescein-labeled 3′UTR probe, recognizing the long variant. Slides were then treated with anti-fluorescein-POD (1:500, Roche, 11426346910) and developed with the fluorescein substrate kit (1:200, NEL741001KT, PerkinElmer). Nuclei were counterstained with a mounting media containing DAPI (Vector, H1200). The specificity of the labeling was confirmed by omitting the riboprobes and obtaining negligible signal.

Results were examined using a Leica SP5 confocal microscope, equipped with excitation lasers 488, 520 and 570 nm, to image, respectively, DAPI (cell nuclei), fluorescein (common mRNA of *Pes1* and 3′UTR long form mRNA of *Gng2*) and Cy3 (3′UTR long form mRNA of *Pes1* and common mRNA of *Gng2*). The objective lens was set at 20× and at 40× magnification. When used, the Z-stacks was 1 μm thickness. The pinhole, photomultiplier tube gain and contrast settings were constant for all image stacks acquired from a slide. For the profiling of the fluorescent signal, the ImageJ plug in was used. A rectangular ROI was used to detect the intensity values from Ventricular- to Subventricular/Mantle Zone of the MGE and from the Ventricular/Subventricular Zone to the Cortical plate (CP) of the embryonic cortex. Significance was calculated with the *t*-test.

## Results

### RNAseq of Proliferating vs. Differentiating ANS Cells

Changes in 3′UTR length due to APA have been documented during embryonic brain development (Hilgers et al., 2012; Miura et al., 2013; Tallafuss et al., 2015), but a systematic examination of this phenomenon during neuronal differentiation, in particular in GABAergic neurons, is lacking. We decided to apply RNAseq to compare transcriptomes of proliferating vs. differentiating ANS cells, a valid model of inhibitory neuron differentiation (Pollard et al., 2006; Onorati et al., 2011; Paina et al., 2011). We used as reference condition proliferating ANS cells, maintained in complete growth medium, and compared them with ANS cells induced to differentiate by: changing to medium D1 for 2 days (T1, reduced proliferation and early commitment); medium D1 for 2 days followed by medium B for 2 days (T2, beginning of differentiation); T2 procedure followed by additional 10 days in medium B (T3, overt differentiation). In all cases three independent replicas were used, with the exception of T3 for which only two samples were obtained (Supplementary Table S2).

To identify the DEGs, we used a FDR cutoff of 0.05. We detected 2334 downregulated genes in T0 vs. T1, 3007 in T0 vs. T2 and 1242 in T0 vs. T3. Conversely, we detected 2483, 2974 and 981 upregulated genes in, respectively, the comparisons T0 vs. T1, T0 vs. T2 and T0 vs. T3. In all comparisons, 895 DEGs were always downregulated and 868 DEGs were always upregulated, indicating a significant uniformity of the differentiation process at the transcriptional level (data deposited at GEO, GSE119073). We also verified the upregulated expression of neuronal and GABAergic markers, and confirmed previously reported data, indicating a proper differentiation of these cells towards the inhibitory lineage (Supplementary Figure S1).

**Figure 1:**
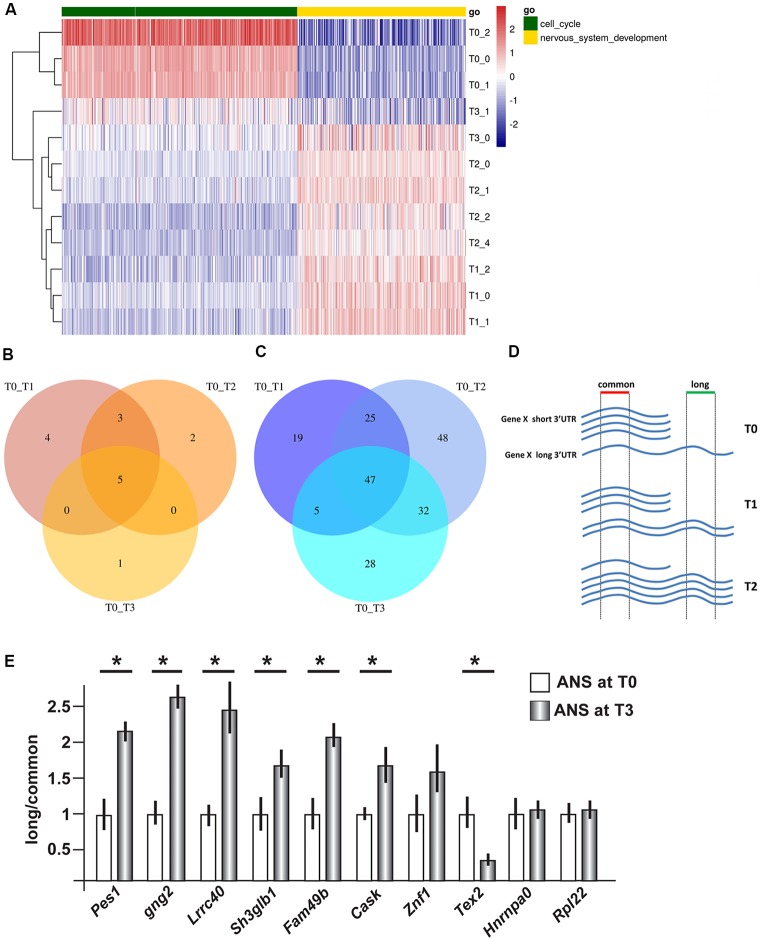
Gene expression profiling and 3′ untranslated region (3′UTR) length in differentiating adherent neural stem (ANS) cells. RNAseq data were obtained from ANS cells at T0 (proliferating), T1, T2 and T3 (three timepoints of GABAergic differentiation). **(A)** Cluster analyses and heat Map of differentially expressed coding mRNAs, relevant to the control of cell cycle exit (in green) and early neuronal differentiation (in yellow). The color-code of the raw Z-scores is shown on the right. **(B,C)** Venn diagrams summarizing the number of transcripts showing shortening **(B)** or lengthening **(C)** during inhibitory neuron differentiation, comparing proliferating (T0) with differentiating (T1, T2, T3) ANS cells. **(D,E)** Technical validation of 3′UTR lengthening. A scheme illustrating the strategy used to quantify the relative abundance of the long vs. common forms of 3′UTR is shown in **(D)**. Ten selected 3′UTRs were examined by Real-Time qPCR analysis (in **E**). Eight of them showed changes in the relative abundance consistent with the RNAseq data. See also Supplementary Tables S3A,B for a full list. *p < 0.05.

We then carried out functional classification analyses on the DEGs identified in all comparisons. Among the genes expressed at higher levels at T0 we identified a significant enrichment of cell cycle and proliferation genes while among those upregulated in differentiating ANS (T1, T2 and T3 vs. T0) we identified a significant enrichment of neuronal differentiation genes, as expected (Figure 1A, Supplementary Figures S2, S3).

Next, we carried out a cross-check of our data with datasets related to cell proliferation and neural differentiation, using GSEA. We identified positive correlations between genes enriched in proliferating ANS cells and cycling/mitotic genes, and with a dataset from tailless-like (TLX)-regulated genes (TLX is a nuclear receptor/transcription factor implicated in the control of proliferation/differentiation step of neural stem cells; Niu et al., 2011; Sun et al., 2011; Islam et al., 2015; Ni et al., 2015; Supplementary Figure S3). Interestingly we also detected an anti-correlation with a dataset related to oligodendrocyte differentiation (Supplementary Figure S3), possibly indicating that neuronal differentiation requires repression of a cognate but distinct cell fate, in this model.

### Alternative PolyA+ Site Usage and Abundance of Transcript Variants With Longer/Shorter 3′UTR in Differentiating ANS Cells

Using the Bioconductor package roar (Grassi et al., 2016), we examined the obtained RNAseq data searching for transcripts showing a significant switch in the use of the proximal or the distal polyA+ site during differentiation. From now on, we will refer to these transcripts as “shorter” and “longer” variant, respectively. Similar to the differential expression at mRNA level, the 3′UTR analysis yielded highly uniform results for the three different time points comparisons, with a notable prevalence of elongation of the 3′UTRs during the differentiation process (Figures 1B,C). Indeed, we identified only five 3′UTRs that switched to the use of the distal polyA+ site, and 47 3′UTRs that switched to the use of the proximal polyA+ site, in proliferating cells (T0) compared to differentiated ones. A list of the common shortened and elongated genes is provided in Supplementary Tables S3A,B. These results indicate a shift in the use of distal PAS during the inhibitory neuron differentiation, *in vitro*.

We then examined the function of all classes of those mRNA showing APA, using standard functional enrichment procedures (based on GO and GSEA; Sergushichev, 2016). Interestingly, two out of the five genes showing shortened 3′UTR encode tubulin subunits, which may correlate with the increased tubulin expression that characterize neuronal differentiation (Guo et al., 2010). Among the 47 common elongated mRNAs, we did not observe specific enrichments in functional classes directly implicated in neuronal differentiation after multiple test correction. Instead, we observed that the major biological processes in which these genes are significantly enriched were “RNA processing” and various “housekeeping” functions (Supplementary Table S4).

Nonetheless several of these genes could play an important functional role in the switch between proliferation and differentiation. For instance, it is well known that subtle modifications of Mapk1 activity, resulting in transient vs. sustained activation, may be a determinant of the choice between neuronal proliferation and differentiation (Marshall, 1995). Rab23 regulates brain development antagonizing the Sonic Hedgehog pathway (Lim and Tang, 2015) which plays a crucial role in specifying the fate of cortical interneurons (Vazin et al., 2014). Modulation of the Smad-repressor Pmepa1 (Liu et al., 2011), of Ppp2r1b (Yeh et al., 2007) and of Rbx1 (Carrano and Pagano, 2001) may contribute to inhibit cell cycle progression. Reduced expression of Serf2, previously identified as BE301622, induces neural stem cells differentiation (Wen et al., 2007), Znrf1 modulates axon extension (Yoshida et al., 2009) and presynaptic development (Araki and Milbrandt, 2003) while Nlgn1 and Cask are implicated in differentiation of inhibitory neurons and synapses (Pettem et al., 2013; Tanabe et al., 2017) by binding to neurexin family proteins (Bang and Owczarek, 2013). Importantly, mutations of *Cask* (Moog et al., 1993), *Hrnrpu* (Bramswig et al., 2017) and *Gnb1* (Petrovski et al., 2016) genes have been associated to neurodevelopmental syndromes characterized by intellectual disability and epilepsy. These results further support a functional link between the alternative polyA+ site usage and neuronal differentiation.

### Experimental Validation of Shorter and Longer 3′UTRs Variants

In order to carry out an independent validation, we selected a number of mRNAs differentially elongated or shortened, for qPCR analyses. The choice was operated ranking them using the roar values and then from the top ones applying different filters, namely: FPKM > 1 at T0 and T3, PRE and POST regions not overlapping with other transcripts and the uniqueness of the designed primers. We then proceeded to validate changes in 3′UTR length by qPCR, on independent RNA samples from proliferating and differentiating ANS cells. The general experimental strategy is shown schematically in Figure 1D. In differentiated ANS cells, one of the transcripts (*Tex2*) showed shortening, six of the transcripts (*Pes1, Gng2, Lrrc40, Sh3glb1, Fam49b* and *Cask*) showed lengthening, while three transcripts (*Znf1*, *Hnrnpa0* and *Rpl22*) remained unchanged (Figure 1E). Thus, for seven out of ten 3′UTRs examined, the RT-PCR results confirmed the expected change in abundance of the longer vs. the common transcript detected by RNAseq. This result strengthens the validity of the RNAseq results and of our bioinformatics pipeline to detect shorter/longer 3′UTRs.

### 3′UTR Variants of *Pes1* and *Gng2* mRNAs Are Enriched in Differentiation Regions on the Cortex and Ganglionic Eminence of the Mouse Brain

**Figure 2:**
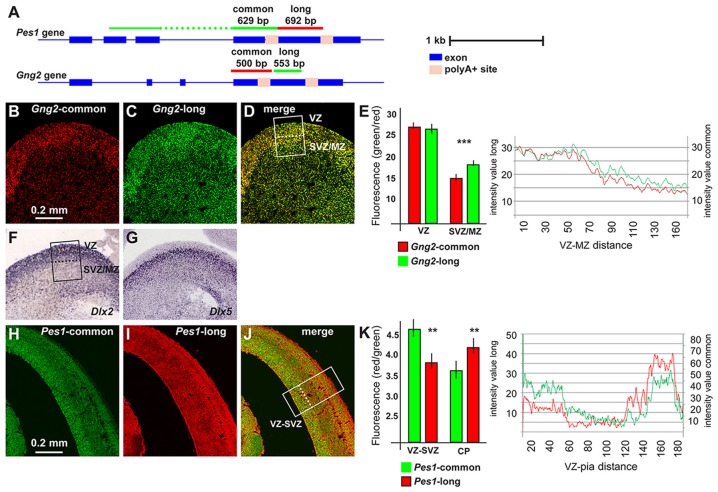
*Pes1* and *Gng2* transcript variants in the mouse embryonic brain. Detection and localization of variants of *Pes1* and *Gng2* transcripts in the mouse embryonic cortex and ganglionic eminence (GE) of the forebrain, to reveal differential abundance in proliferating vs. differentiating regions. **(A)** Scheme showing the *Gng2* and *Pes1* gene organization, the location of the alternative polyA+ sites and of the probes used to detect all variants (common) or the long-variants. Scale is shown on the right. **(B–D)** Coronal sections of normal mouse embryonic forebrain at the age E14.5 subjected to two-colors fluorescent *in situ* hybridization (FISH) to determine the localization and relative abundance of variants of the *Gng2* mRNAs. At least 10 sections were stained, obtained from two brain specimens. Representative images of two-colors FISH to detect all variants (red, in **B**) or the long variant (green, in **C**) *Gng2* transcripts in the basal brain primordium. A merged image is shown in **(D)**. **(E)** Histogram and profile of the relative fluorescent intensity of the images in **(B–D)**, from the area indicated in (**D**; white rectangle, subdivided in ventricular zone(VZ) and subventricular/mantle zone (SVZ/MZ). At least two areas/slide were considered. The results relative to the VZ or the SVZ/MZ are shown as histogram. *Gng2*-all variants is indicated with red bars and lines, the *Gng2*-long variant is indicated with green bars and lines. Note the relative increase in the relative fluorescent detected in the SVZ/MZ area. **(F,G)** Images of the expression and localization of *Dlx2* **(F)** and *Dlx5* **(G)** mRNAs by ISH (from www.genepaint.org) marking, respectively, proliferating progenitors and committed/early differentiating GABAergic neurons. The position of the VZ and SVZ/MZ is indicated in **(F)**. **(H–J)** Representative images of two-colors FISH to detect all variants (green, in **F**) or the long variant (red, in **G**) *Pes1* transcripts in the cortical primordium. A merge image is shown in **(H)**. **(K)** Histogram and profile of the relative fluorescent intensity of the cortical primordium, from the areas indicated in (**H**; (white rectangle, subdivided inVZ/SVZ and cortical plate (CP)). At least two areas/slide were considered. The results relative to the VZ/SVZ or the CP are shown as histogram. *Pes1*-all variants are indicated with green bars and lines, *Pes1*-long variant is indicated with red bars and lines. Note the relative increase in the relative fluorescence detected in the CP. Asterisks indicate statistical significance, **p < 0.01, ***p < 0.001.

For a further validation of the lengthening of 3′UTR during neuronal differentiation, and in order to confirm the differential abundance of the longer form in differentiating neurons, we chose to examine the expression of long-variants of *Gng2* and *Pes1* transcripts *in vivo*. We collected coronal sections of the embryonic mouse brain (ages E14.5 and E18.5) and determined the localization and level of expression of the 3′UTR longer vs. all transcript variants of these two genes, by FISH. For each gene two probes were prepared: one that specifically detects the longer variant, labeled in with one fluorescence, and the other detecting all variants of the same, labeled with a different fluorochrome (Figure 2A, for probes info see Supplementary Table S1C). With these probes we carried out two-colors RNA:RNA FISH on sections of embryonic mouse brains, according to published procedures (Grosso et al., 2015) and quantified confocal images of the ganglionic eminence (GE) and the primordiun of the dorsolateral cortex.

In the GE we observed expression of the all-variant *Gng2* transcript in the Ventricular-, the Subventricular- and the Mantle Zone (VZ, SVZ, MZ), with a preference in the VZ, the location in which interneuron progenitors actively proliferate (Hu et al., 2017). Instead the probe specific for the long-*Gng2* transcript reveals a higher relative signal in the SVZ/MZ, where post-mitotic early-differentiating neurons are present (Figures 2B–D). The fluorescent intensity of the longer and the all-variant probes was quantified along the thickness of the GE and plotted as a function of the position. A significant difference (*p* < 0.001) in the relative fluorescence in the SVZ/MZ is documented (Figure 2E). These results confirm that the expression of the longer *Gng2* variant is enriched in zones of differentiating inhibitory neurons. As a further comparison, we added two high-resolution images of mRNA expression by *in situ* hybridization (from www.genepaint.org) showing the relative expression pattern of *Dlx2* and *Dlx5* in the VZ and SVZ/MZ of the mouse embryonic GE (Figures 2F,G). There two mRNAs are excellent markers for proliferating neuronal progenitors (*Dlx2*) and early differentiating progenitors (*Dlx5*) of the GABAergic lineage (Eisenstat et al., 1999; Stühmer et al., 2002; Perera et al., 2004).

We then extended this observation and examined the expression of the *Pes1* transcript variants in the mouse embryonic cortex at the age E14.5, as above. The FISH experiments revealed a strong expression of the common *Pes1* form throughout the cortex thickness, with an increased abundance in the VZ/SVZ and in the CP. Instead, the probe detecting only the longer *Pes1* transcript shows reduced relative expression in the VZ/SVZ and increased relative expression in the CP (Figures 2H–J). The relative fluorescent intensity was determined and plotted as above, and documented a significant difference (*p* < 0.001) in the abundance of the longer vs. common *Pes1* transcripts in the VZ/SVZ compared to the CP (Figure 2K). The VZ and SVZ regions of the embryonic cortex at this age harbors the neural progenitors and the proliferating neuroblasts, while the CP corresponds to the position of post-mitotic early differentiated neurons. Globally, these data indicate that differentiating neurons of both the basal and the cortical embryonic brain switch to the use of distal (or proximal) APA sites, at least for the *Pes1* transcript.

### Expression of *Elavl* Genes and Other RBPs Involved in APA Choice During Neural Differentiation

To get hints on the possible molecular drivers of alternative polyA+ site usage in our model of ANS cell differentiation, we examined RNAseq-based profiling data focusing on the expression of *Elavl* and other RBP potentially involved in APA choice. Elav proteins, first identified in *Drosophila* and then in mammals (named Elavl1-4, also known as HuR, HuB, HuC and HuD, respectively; Good, 1995), participate in the formation of a complex that, in synergy with the promoter state of a transcribed gene, operates the choice of polyA+ sites (Oktaba et al., 2015). *Drosophila* Elav protein is mainly expressed in the insect neurons and has been shown to participate in their differentiation and axon guidance (Simionato et al., 2007; Colombrita et al., 2013). In mammals, Elavl2-4 proteins are also mainly expressed in neurons (Ogawa et al., 2018), but their functions is still to be explored.

**Figure 3:**
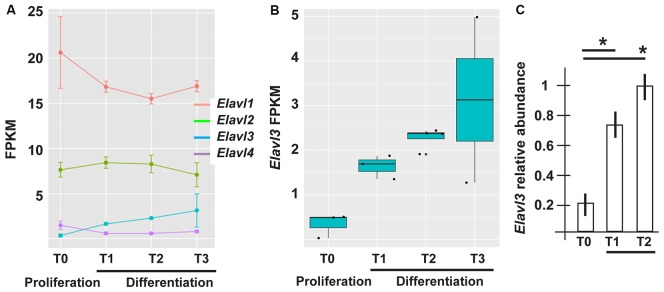
Differential expression of RNA binding protein (RBP) of the Elavl family in differentiating ASN cells. **(A)** Expression of *Elavl1*, *Elavl2, Elavl3* and *Elavl4* RBPs in proliferating (T0) vs. differentiating (T1, T2, T3) ASN cells, by RNAseq analysis. **(B)** Plotting of the same data as in **(A)** showing differential expression of *Elavl3* mRNA in proliferating vs. differentiating ASN cells, by RNAseq analysis. **(C)** RealTime qPCR analyses on independent RNA samples to quantify the relative abundance of *Elavl3* mRNA in ASN cells at T0, T1 and T2. Results are expressed as relative abundance, T2 is made = 1. *p < 0.05.

The mRNAs for RBPs known to play role in the polyadenylation and choice of polyA+ site, such as members of the Cleavage Factor I family (Yang et al., 2011; Martin et al., 2012) did not show differential expression across the time points of ANS differentiation (data not shown). On the contrary, in our RNAseq data *Elavl3* mRNA showed an interesting pattern: upregulated (log2 fold changes +2.24 and 2.01) in T2 and T3 vs. T0, and (+1.56) in T1 vs. T0 (Figures 3A,B). Real-Time qPCR analysis of *Elavl3* expression on independent samples of proliferating vs. differentiating ANS cells confirmed this finding (Figure 3C). No other member of the *Elavl* family was found to be differentially expressed, with the exception of *Elavl4* that was downregulated in T1 and T2 vs. T0 (Figure 3A). This may suggest that Elavl3 could participate in APA during neuronal differentiation. This result is in accordance with previous observations indicating that *Elavl2* is expressed in early neuronal progenitors and in mature neurons, while *Elavl3/4* expression begins slightly later, during cortical neuron development (Yano et al., 2015).

To obtain further evidences of a role of Elavl3 in the lengthening process and in order to overcome the limitations of seeking binding sites with *in silico* approaches, we used a collection of Elavl3 binding sites experimentally determined via HITS-CLIP methods in the mouse brain (Ince-Dunn et al., 2012). We detected a significant overlap (Fisher test *p* = 0.0012, 40 out of 47 common lengthened genes overlap an Elavl3 binding site) between Elavl3 targeted transcripts and our list of lengthened genes. This enrichment and the differential expression results suggest that increased Elavl3 expression is a good candidate mechanism to explain 3′UTR lengthening in differentiating inhibitory neurons.

### Silencing of *Elavl3* Affects PolyA+ Site Usage and Reduces the Efficiency of Differentiation

**Figure 4:**
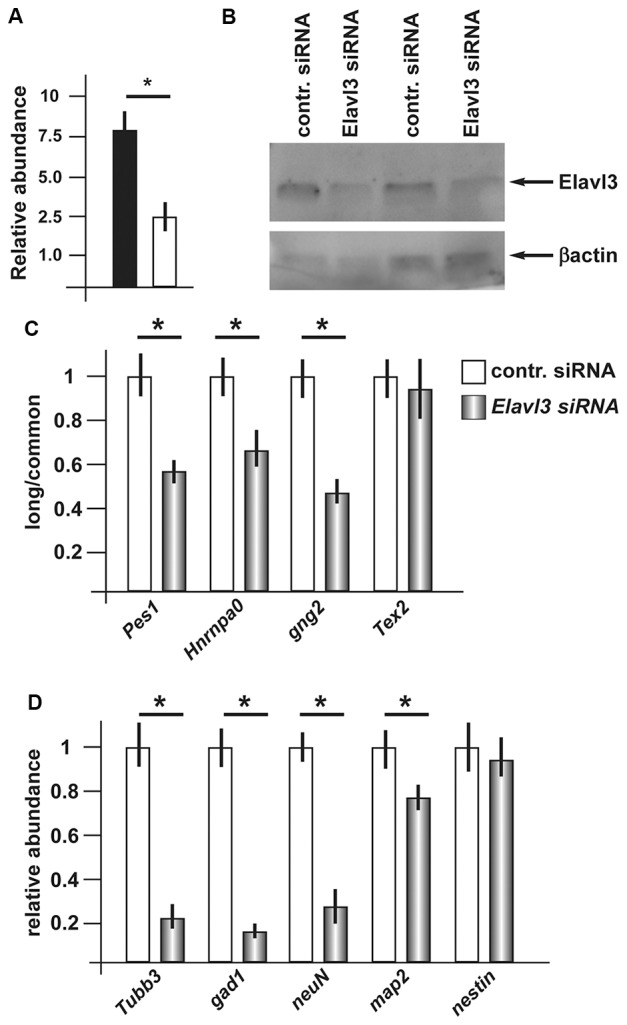
Effect of depletion of Elavl3 in differentiating ANS cells. **(A)** RealTime qPCR analysis to detect the abundance of *Elavl3* mRNA in ANS cells treated with the control (black) or anti-Elavl3 (white) siRNA. **(B)** Western blot analysis of ANS cells treated with the control siRNA or with the anti-Elavl3 siRNA, to confirm the downmodulation of Elavl3 protein (top). Staining with anti-actin on the same blot is used as loading control (bottom). **(C)** Real Time qPCR analysis to detect the relative abundance of the longer vs. the common variants of *Pes1*, *Hnrnpa0*, *Gng2* and *Tex2* mRNAs, comparing siRNA-treated vs. control-treated ANS cells, maintained in differentiation conditions. **(D)** Real-Time qPCR analysis to detect the mRNA abundance of the differentiation genes *GAD1, Tubβ3, NeuN* and *Map2* in ANS cells treated with the control (black bars) or anti-*Elavl3* (open bars) siRNA. *p < 0.05.

Having observed consistent increase in *Elavl3* mRNA, and considering its known functions, we decided to investigate whether Elavl3 may participate in the mechanism leading to a choice of poly-A site during inhibitory neuron differentiation. To address this possibility, we applied Accell siRNA oligonucleotide targeting Elavl3 (see “Material and Methods” section and Supplementary Table S1A) to differentiating ANS cells. We first verified that treatment with this reagent caused a depletion of the endogenous *Elavl3* mRNA and protein, by Real-Time qPCR and Western blot analyses, respectively (Figures 4A,B). Next, we determined the relative abundance of the longer vs. the common variants of a number of selected mRNAs, comparing siRNA-treated vs. control-treated ANS cells, maintained in differentiation conditions. For this analysis, RT-PCR was used according to previous publications. The following transcripts were chosen: *Pes1*, *Hnrnpa0*, *Gng2* and *Tex2*. In three out of four of these (*Pes1*, *Hnrnpa0* and *Gng2*) we detected a clear shift of the relative abundance, towards the usage of the proximal polyA+ site at the expenses of the distal site in cells depleted of Elavl3, as compared to control-treated ANS cells (Figure 4C). This result indicates that Elavl3 participates in the polyA+ site selection during neuronal differentiation.

Next, we examined the effect of silencing of *Elavl3* on the efficiency of ANS cell differentiation. As above, endogenous *Elavl3* was depleted by treating differentiating ANS cells with siRNA Accel, followed by measurement of the abundance of four known differentiation markers including *GAD1, Tubβ3, NeuN* and *Map2*, by Real-Time qPCR. While *Tubβ3, NeuN* and *Map2* code for pan-neuronal differentiation genes, *GAD1* codes for the GABA-synthesizing enzyme GAD67, specific of inhibitory neurons. In addition, the expression of the neural progenitor cell marker *nestin* was determined, as further control. As compared to control-treated ANS cells, Elavl3-depleted cells showed a significant reduction in the expression of *Tubβ3* and *Gad1* mRNAs, while the global expression of *nestin* remained unchanged (Figure 4D). These results indicate that upon downmodulation of Elavl3, ANS cells undergo inefficient or delayed differentiation, while the progenitor properties are not affected.

## Discussion

The transcriptome and its dynamic changes have turned out to be extremely complex. Such complexity has the reasonable significance of increasing the information-content of RNA coding sequences, of fine-tuning RNA translation and stability in specific sub-cellular compartments, and engaging in RNA:RNA networks cross-regulating each other (Loya et al., 2010). These features are well adapted to participate in “defining” the extreme variability and complexity of neuronal morphology, connectivity and activity.

We have examined the transcriptome of a cellular model of basal forebrain neural progenitors induced to differentiate towards the GABAergic fate. We found a clear shift in the usage of distal polyadenylation sites in differentiating cells compared to proliferating ones for a significant number of genes. This general result is in agreement with previous reports showing a preferential usage of distal sites in differentiated cells during embryonic development (Ji Z. et al., 2009). A shift from short to longer 3′UTR isoforms have been observed during neural cell types differentiation, such as cerebellar granule cells (Jereb et al., 2018). However, to our knowledge, the progenitors of the inhibitory neurons have not been studied and this is the first report in which APA and a role of Elavl3 is documented in differentiating mammalian inhibitory neurons.

We examined the function of all classes of those mRNA showing APA, using GO analyses. We failed to observe specific enrichments in functional classes that were previously and directly implicated in neuronal differentiation. Instead, we observed that the major biological processes in which these genes are significantly enriched were “RNA processing” and various “housekeeping” functions. This finding is in line with a previous study in which the authors showed that genes that are ubiquitously transcribed in all body tissues tend to harbor more than one polyadenylation site, while genes that harbor only one polyadenylation site are expressed in a clear tissue-specific manner (Lianoglou et al., 2013; Mayr, 2016). Hence, widely expressed genes may use elements located in their alternative 3′UTRs to achieve tissue-specific expression or function (Lianoglou et al., 2013; Berkovits and Mayr, 2015), while tissue specificity might be more dependent on cis-acting promoter elements and regulation of gene transcription at promoter level.

Although no significant functional enrichments for the shorter vs. longer 3′UTRs was detected, nonetheless two (of five) genes of the GO term “neurexin family protein binding,” *Nlgn1* and *Cask* were differentially polyadenylated with an elongated 3′UTR. Neurexins are presynaptic cell adhesion molecules implicated in various neuronal processes, including the differentiation, maturation, stabilization, and plasticity of both inhibitory and excitatory synapses (Bang and Owczarek, 2013). Recent data further indicate that neurexins are implicated in the differentiation of inhibitory synapses (Pettem et al., 2013; Tanabe et al., 2017), thus supporting a functional link between the alternative polyA+ site usage and neuronal differentiation.

The functional significance of APA at the transcriptome level remains speculative. Longer 3′UTR variants of coding (or non-coding) transcripts include sequences not present in the shorter variant. Hence, longer variants could represent gain-of-function variants that engage in miR-based regulations that do not influence the shorter variant. We have searched for miR target sequences significantly enriched in the extra sequence present in the lengthened 3′UTRs of transcripts showing APA, and we detected miR-216, miR-15, miR-329, miR-19, miR-146 e miR-539. Thus we may hypothesize that the use of distant polyA+ site would potentially place the longer transcripts under a negative control by these miRs, or conversely, that the shorter variants may escape regulation by these miR in proliferating ANS cells. We have examined the profile of expressed miR in differentiating vs. proliferating ANS cells: while none of the miR indicated above were up- or downregulated, miR-15 and miR-19b were found to be stable and highly expressed in these cells (data not shown) and therefore may represent putative new regulators of differentiation-related elongated 3′UTRs. This possibility will be explored in future works.

Another possible role of global lengthening of the 3′UTR could be in the ceRNA network regulation. Indeed, it has recently been reported that global shortening due to APA represses tumor suppressor genes via a ceRNA-based mechanisms (Park et al., 2018). Lengthening of the 3′UTR, by contrast, should derepress tumor suppression activities.

At the molecular level, the full mechanism controlling the choice of the polyA+ site has been clarified only in part, and certainly involves a protein complex which includes members of the Elavl and CP families (Yang et al., 2011; Hilgers et al., 2012; Martin et al., 2012; Schönemann et al., 2014; Oktaba et al., 2015; Zhu et al., 2018). Elav in *Drosophila*, and Elav-like proteins in vertebrates (Elavl1-4, also known as Hu autoantigens associated with a multi-systemic neurological disorder named paraneoplastic encephalomyelopathy) have been shown to play a key role in APA and to be required for neural commitment. In *Drosophila* Elav is detected in the nuclei of early embryonic neurons and the absence of Elav in *Drosophila* causes embryonic lethality and failure of eye formation, via altered RNA processing (Colombrita et al., 2013; Zaharieva et al., 2015). In the mammalian neocortex, Elavl1 determines the temporal pattern of translation and polysome assembly (Kraushar et al., 2014). Here we show that in a model of (mammalian) neural progenitors the depletion of Elavl3 causes a shift in favor of proximal APA usage of some selected transcripts and delayed neuronal differentiation. Importantly, when Elavl3 was depleted we observed the downregulation of neural markers (*Gad1* and *TubβIII*), suggesting delayed GABAergic differentiation.

In addition to APA regulation, *Drosophila* Elav interacts with paused RNAPol-II at promoter regions (Hilgers, 2015; Oktaba et al., 2015). It has long been known that mRNA maturation is a co-transcriptional event, however only recently a molecular link between the promoter status (paused Pol-II) and polyA+ site usage has been unravelled. Indeed, Elavl proteins link these two processes, thus Elavl proteins are not simply RBP and RNA-modifying proteins but, in addition, may couple transcription at the promoter level with specific variants of the mature RNA. Due to the high conservation of *Elav*-related genes and proteins, it is possible to speculate that the mechanism identified in *Drosophila* could be true also in mammalian cells, but no clear evidence of this is currently available.

Unlike *Drosophila* Elav, mammalian Elavl1-4 are shuttle proteins that are detected both in the cytoplasm and in the nucleus (Colombrita et al., 2013). This may indicate that Elavl proteins play more complex functions, in addition to APA regulation: 3′UTR-bound Elavl3 protein could act as scaffold to recruit a protein complex containing another protein of the same class, such as Elavl1/HuR (Kraushar et al., 2014). During translation the scaffold function of 3′UTRs facilitates binding of proteins to nascent proteins to direct their transport or function and this role can be regulated by APA.

The function of Elavl3 has been examined *in vivo* by the generation and phenotype analysis of *Elavl3* KO mice. These animals show neurological defects and specifically altered control of the glutamatergic system and altered neuronal excitability (Ince-Dunn et al., 2012). The authors also describe a reduction of glutamate neurotransmitter, which accompanies an increased propensity to undergo epileptic seizures. These observations are suggestive of an impairment in GABAergic circuits. The same mice have be recently re-examined for fine neuronal phenotypes. Elavl3 is essential for the maintenance of Purkinje neuron axons and regulates polarity of Purkinje neurons through the alternative splicing an embryo-specific exon in Ankyrin G (Ogawa et al., 2018), via a yet unknown mechanism. The participation of *Drosophila* Elav and related RBPs in splicing regulation, in addition to the control of APA, has been previously shown (Zaharieva et al., 2015).

We have shown that *Elavl3* mRNA expression is significantly increased during GABAergic differentiation of ANS cells, and we have shown that this process is delayed by *Elavl3* downregulation. Together these finding suggest that Elavl3 is a key player in GABAergic interneurons. Interestingly Pollen et al., 2015 analyzed gene expression across single cells during human cortical neurogenesis and early neuronal differentiation. They showed that *Elavl4* mRNA is specifically upregulated, together with *NeuroD2* and *-6* genes, at the passage between intermediate progenitors and early differentiating neurons in human cortex. The biological significance of this increase is unknown, and especially the association of this with APA cannot be assessed, as the single cell profiling data does not easily consent the examination of 3′UTR of transcripts. Given the strong homology between Elavl3 and -4, we can speculate that Elavl3 and -4 may play similar functions in inhibitory and excitatory neurons, respectively, during cortical development, or that alternatively mice and human use Elavl3 and -4 for the same function.

Variations in sequences at the 3′UTR of transcribed RNAs are relevant for neurodevelopmental disorders. A specific and direct involvement of Elavl1 (i.e., HuR antigen) has been shown in cases of Fragile X Syndrome, in which mutations in the 3′UTR of *FMR1* abrogate a Elavl1/HuR binding site (Collins et al., 2010; Suhl et al., 2015). In addition to this, several 3′UTR variants have been associated to neurological and cognitive disorders, including Rett syndrome, schizophrenia and autism spectrum disorders, via mechanisms of altered RNA stability and/or altered miRNA repression (Wanke et al., 2018). Considering that sequences in the 3′UTRs are relevant for synaptic plasticity, neuronal activity and neocortical layering (Aksoy-Aksel et al., 2014; Kraushar et al., 2014; Pilaz and Silver, 2015; Sun and Shi, 2015), variations within the 3′UTRs should have functional consequences for protein expression or localization. These variants, being subtle, are more likely to contribute to neurodevelopmental disorders in a polygenic, low risk, fashion.

Finally, a recent cohort analysis of the human genome shows that mutations and SNPs in RBPs are associated with various neurological disorders (Yano et al., 2015). Misregulation, mutations or sequestration into nuclear or cytoplasmic inclusions of RBPs have been linked to fragile-X syndrome, autism spectrum disorders, spinal muscular atrophy, amyotrophic lateral sclerosis and frontotemporal dementia. In particular, Elav proteins are associated with Paraneoplastic Encephalomyelopathy/Paraneoplastic Sensory Neuropathy and Parkinson’s disease (Ravanidis et al., 2018). Elavl2 (SNP ID, rs10491817) is associated with schizophrenia, particularly in Asian populations (Yamada et al., 2011). Newly emerged technologies, able to assess transcriptome-wide RBP-protein interactions *in vivo*, combined with classical genetics methods, may provide new insight into Elavl proteins, not only with respect to their neurodevelopmental functions, but also their roles in diseases.

In conclusion, with this study we highlight the profound changes in 3′UTR length in early steps of differentiation of basal-type neural progenitors, committed to GABAergic differentiation. We found evidence that Elavl3 protein plays a prominent role in this process and participates to determine the efficiency of GABAergic terminal differentiation. Alternative usage of polyA+ sites suggests that yet another regulation takes place at the cotranscriptional level, which contributes to transcriptome complexity and neuronal specification.

## Ethics Statement

This study was carried out in accordance with the recommendations of the the Internal Ethical Committee of the School of Medicine. The protocol was approved by the Italian Ministry of Health—General Authority of Animal Health and Veterinarian Medicine (authorization number 51/2018-PR).

## Author Contributions

EG, RS, PP, FDC and GM conceived and designed the experiments. EG, RS and AU performed the experiments. EG, RS, AU, SO, FN, PP, FDC, UA and GM analyzed the data. EG, AG, SO, FN, LC, UA and PP contributed reagents, materials and analysis tools. EG, RS, AU, FDC and GM wrote the article.

## Conflict of Interest Statement

The authors declare that the research was conducted in the absence of any commercial or financial relationships that could be construed as a potential conflict of interest.

## Abbreviations

ANS: Adherent Neural Stem
APA: Alternative polyadenylation
DEG: Differentially Expressed Gene
GAD: Glutamic Acid Decarboxylase
3′UTR: 3′Untranslated Region
RBP: RNA binding protein
miR: microRNA.
