# Supplementary material for: Choice of Alternative Polyadenylation Sites, Mediated by the RNA-Binding Protein Elavl3, Plays a Role in Differentiation of Inhibitory Neuronal Progenitors
Source: Front Cell Neurosci. 2019 Jan 10;12:518. doi: 10.3389/fncel.2018.00518 (PMC6338052; doi:10.3389/fncel.2018.00518)
Supplement: Supplementary file 1 [file Table_1.DOCX]

**Supplementary Table 1A. Sequences of the Accel siRNA oligonucleotides to target Elavl3**

Target sequence Mol. Wt. (g/mol)

5’ GCAAGUUGGUUCGGGAUAA 13545,9
5’ GGAGGGAACAGGUUUAAAA 13473,9
5’ CGUCUUGGUUUGGUUCAUG 13605,4

5’ GCACAAGGCCUGAGCAUUU 13566,6

**Supplementary Table 1B. Sequence of oligonucleotides used for RealTime qPCR**

*Hnrnpa0 Pre* F 5’ AGAAAACGCTGTAGCCAGGT

R 5’ CGGGAGTGAACTTGCTGAT

*Hnrnpa0 post* F 5’ CAAAGAGATGGATGCACAGG

R 5’ TCCTGTTTGCCCAGTTAGTG

*Gng2 Pre* F 5’ CTTAAAGGATGGCGCAGAAG

R 5’ ACAAGCCAGGAAGCTGGTAG

*Gng2 Post* F 5’ AAAGATGCGCACATCACATT

R 5’ ATTCCCCTGTCTCTGACTCG

*Pes1 Pre* F 5’ CTTTCGGCCTTGGAGCAG

R 5’ CTCACAGCATCATCATGGGC

*Pes1 Post* F 5’ TAGGACTCCCGAGTTCAAGG

R 5’ CTGAGCTGAAACAGGAACCA

*Tex2 Pre* F 5’ TCAAGAGGCAGGAAGTGGAG

R 5’ CTGAAAGCTCGGCCAAAACT

*Tex2 Post* F 5’ TGCAGAGAAAGACCAATGAAC

R 5’ TTTGACCATTCCAGTCAGTG

*Lrrc40 Pre* F 5’ATCAGGTTGGCTCTGTGGAC

R 5’GGTACTCAGGCAGGGATTCG

*Lrrc40 Post* F 5’CCCGGGTACTGCACTTATGG

R 5’TGCCCTCAGTAATTTGTGGGA

*Sh3glb1 Pre* F 5’CAGGAAGGCTAGGGTCCTCT

R 5’TGGGTAACCTCTCGTGGTCT

*Sh3glb1 Post* F 5’TGCAGAACACTGGGGACAAA

R 5’AGACAGACTGCATCCACTGC

*Fam49b Pre* F 5’GTGTAGAAGGTCTTCTCAATGCTC

R 5’AGTGGGAAGAGGAACAGAGGA

*Fam49b Post* F 5’AGCCCAAGGCAAAGAAATGTG

R 5’GAGTAAGCAGGAGGCCAGAG

*Cask Pre* F 5’GGACTGCAGAGTTTGCTCCT

R 5’GCTCGACAGCTTCTTCCAGA

*Cask Post* F 5’AATGTTGGAAAGCCAGAGTGC

R 5’TAAGCTGAGGGACACATTCCTTT

*Znfr1 Pre* F 5’CTTCAGGGGGACACGATAGC

R 5’TTTGAGAGGAGTCGGCAAGC

*Znrf1 Post* F 5’GAGGAAGATGAAGAGCCCATCG

R 5’CACAGGGGCCTTGGAAAAATG

*Rpl22 Pre* F 5’GAATGGGAAAGCTGGCAACC

R 5’ACTCTCTTTGCTGTTGGCGA

*Rpl22 Post* F 5’ATGCAATGCTAAAGGGCTCG

R 5’GGGCAGAGGAGAAAGACAGT

*Elavl3* F 5’ AATGGCCTCAAACTGCAGAC

R 5’ GGGAGGCCACTGACATACAG

*Tubb3* F 5’ GAACCTGGAACCATGGACAG

R 5’ GTTGTTGCCAGCACCACTCT

*Gad1* F 5’ CCGTTCTTAGCTGGAAGCAG

R 5’ CCGTTCTTAGCTGGAAGCAG

*Map2* F 5 CAAAGAGATGGATGCACAGG

R 5’ TCCTGTTTGCCCAGTTAGTG

*NeuN* F 5’ GGGAACCTGTCCGAGGAGT

R 5’ TTTATTCAGCCAATTTTCCC

**Supplementary Table 1C. Sequence of oligonucleotides for the generation of FISH probes**

Sequence Amplicon/probe size

***Pes1* common**

F 5’ CTCCCGAGTTCAAGGAAGGT 629 bp

R 5’GTGGAAATGCGAGCCA

***Pes1* long**

F 5’GACCCAGGACACTTGGAAGA 692 bp

R 5’AAAAGCTCACCACCCTCACT

***Gng2*common**

F 5’CAAGTCAGATCTGCCAGGGA 500 bp

R 5’GTCTGCAGGGTCAGATCTCT

***Gng2* long**

F 5’ACACACACCACACAATTCCG 553 bp

R 5’AGCTAAAGAGGGCCATGGTT
