# Supplementary material for: Choice of Alternative Polyadenylation Sites, Mediated by the RNA-Binding Protein Elavl3, Plays a Role in Differentiation of Inhibitory Neuronal Progenitors
Source: Front Cell Neurosci. 2019 Jan 10;12:518. doi: 10.3389/fncel.2018.00518 (PMC6338052; doi:10.3389/fncel.2018.00518)
Supplement: Supplementary file 2 [file Table_2.DOCX]

**Supplementary Table 2**.

Summary of the culture conditions of ANS and the samples used for RNA seq.

| **Time point** | **Culture Conditions** | **Cell Status** | **N. biological replicates** |
| --- | --- | --- | --- |
| T0 | ANS cells in complete growth medium | Full proliferation | 3 |
| T1 | ANS cells 2 days after change in medium D1 | Reduced cell proliferation | 3 |
| T2 | ANS cells 2 days after change in medium B | Cell cycle arrest and early differentiation | 4 |
| T3 | ANS cells 10 days after change in medium B | Overt GAD+ differentiation | 2 |
