# Supplementary material for: Choice of Alternative Polyadenylation Sites, Mediated by the RNA-Binding Protein Elavl3, Plays a Role in Differentiation of Inhibitory Neuronal Progenitors
Source: Front Cell Neurosci. 2019 Jan 10;12:518. doi: 10.3389/fncel.2018.00518 (PMC6338052; doi:10.3389/fncel.2018.00518)
Supplement: Supplementary file 3 [file Table_3.DOCX]

**Supplementary Table 3A. Genes with elongated 3’UTR in differentiated ANS cells**

| **entrez id** | **gene name** |
| --- | --- |
| 269261 | Rpl12 |
| 192167 | Nlgn1 |
| 14702 | Gng2 |
| 70911 | Phyhipl |
| 432530 | Adcy1 |
| 26420 | Mapk9 |
| 223601 | Fam49b |
| 19934 | Rpl22 |
| 64934 | Pes1 |
| 73699 | Ppp2r1b |
| 66970 | Ssbp2 |
| 77134 | Hnrnpa0 |
| 22017 | Tpmt |
| 378702 | Serf2 |
| 26936 | Mprip |
| 67144 | Lrrc40 |
| 54673 | Sh3glb1 |
| 65112 | Pmepa1 |
| 75608 | Chmp4b |
| 19335 | Rab23 |
| 170737 | Znrf1 |
| 18117 | Emc8 |
| 14852 | Gspt1 |
| 17920 | Myo6 |
| 267019 | Rps15a |
| 67443 | Map1lc3b |
| 53379 | Hnrnpa2b1 |
| 12361 | Cask |
| 69367 | Glrx2 |
| 26374 | Rfwd2 |
| 68051 | Nutf2 |
| 230908 | Tardbp |
| 140740 | Sec63 |
| 56491 | Vapb |
| 22032 | Traf4 |
| 20318 | Sdf4 |
| 51810 | Hnrnpu |
| 217057 | Ptrh2 |
| 20024 | Sub1 |
| 14688 | Gnb1 |
| 66467 | Gtf2h5 |
| 26413 | Mapk1 |
| 66870 | Serbp1 |
| 13207 | Ddx5 |
| 68196 | Hsbp1 |
| 56438 | Rbx1 |
| 12313 | Calm1 |

**Supplementary Table 3B. Genes with shortened 3’UTR in differentiated ANS cells**

| **entrez id** | **gene name** |
| --- | --- |
| 22121 | Rpl13a |
| 73710 | Tubb2b |
| 22146 | Tuba1c |
| 12609 | Cebpd |
| 21763 | Tex2 |
