# Supplementary material for: Choice of Alternative Polyadenylation Sites, Mediated by the RNA-Binding Protein Elavl3, Plays a Role in Differentiation of Inhibitory Neuronal Progenitors
Source: Front Cell Neurosci. 2019 Jan 10;12:518. doi: 10.3389/fncel.2018.00518 (PMC6338052; doi:10.3389/fncel.2018.00518)
Supplement: Supplementary file 4 [file Table_4.DOCX]

**Supplementary Table 4. Top enriched gene ontology categories in transcripts showing APA**

| GO:0031281 | positive regulation of cyclase activity | biological_process |
| --- | --- | --- |
| GO:0045761 | regulation of adenylate cyclase activity | biological_process |
| GO:0004707 | MAP kinase activity | molecular_function |
| GO:0051258 | protein polymerization | biological_process |
| GO:0019058 | viral infectious cycle | biological_process |
| GO:0031279 | regulation of cyclase activity | biological_process |
| GO:0051349 | positive regulation of lyase activity | biological_process |
| GO:0031996 | thioesterase binding | molecular_function |
| GO:0036002 | pre-mRNA binding | molecular_function |
| GO:0003924 | GTPase activity | molecular_function |
| GO:0003697 | single-stranded DNA binding | molecular_function |
| GO:0050699 | WW domain binding | molecular_function |
| GO:0030529 | ribonucleoprotein complex | cellular_component |
| GO:0051339 | regulation of lyase activity | biological_process |
