# Supplementary figures and images for: Choice of Alternative Polyadenylation Sites, Mediated by the RNA-Binding Protein Elavl3, Plays a Role in Differentiation of Inhibitory Neuronal Progenitors
Source: Front Cell Neurosci. 2019 Jan 10;12:518. doi: 10.3389/fncel.2018.00518 (PMC6338052; doi:10.3389/fncel.2018.00518)

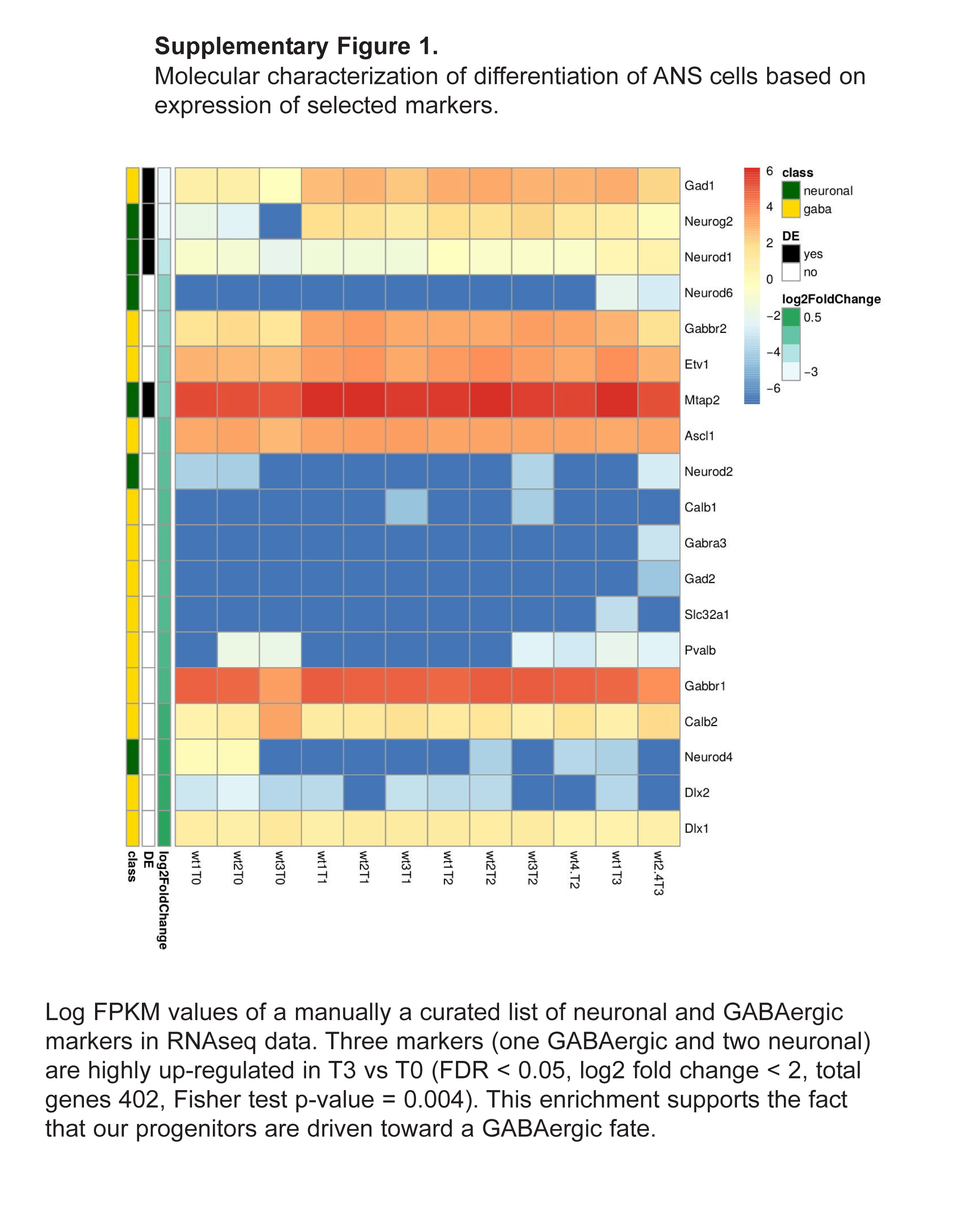

Supplement: Supplementary file 5 [file Image_1.TIF]

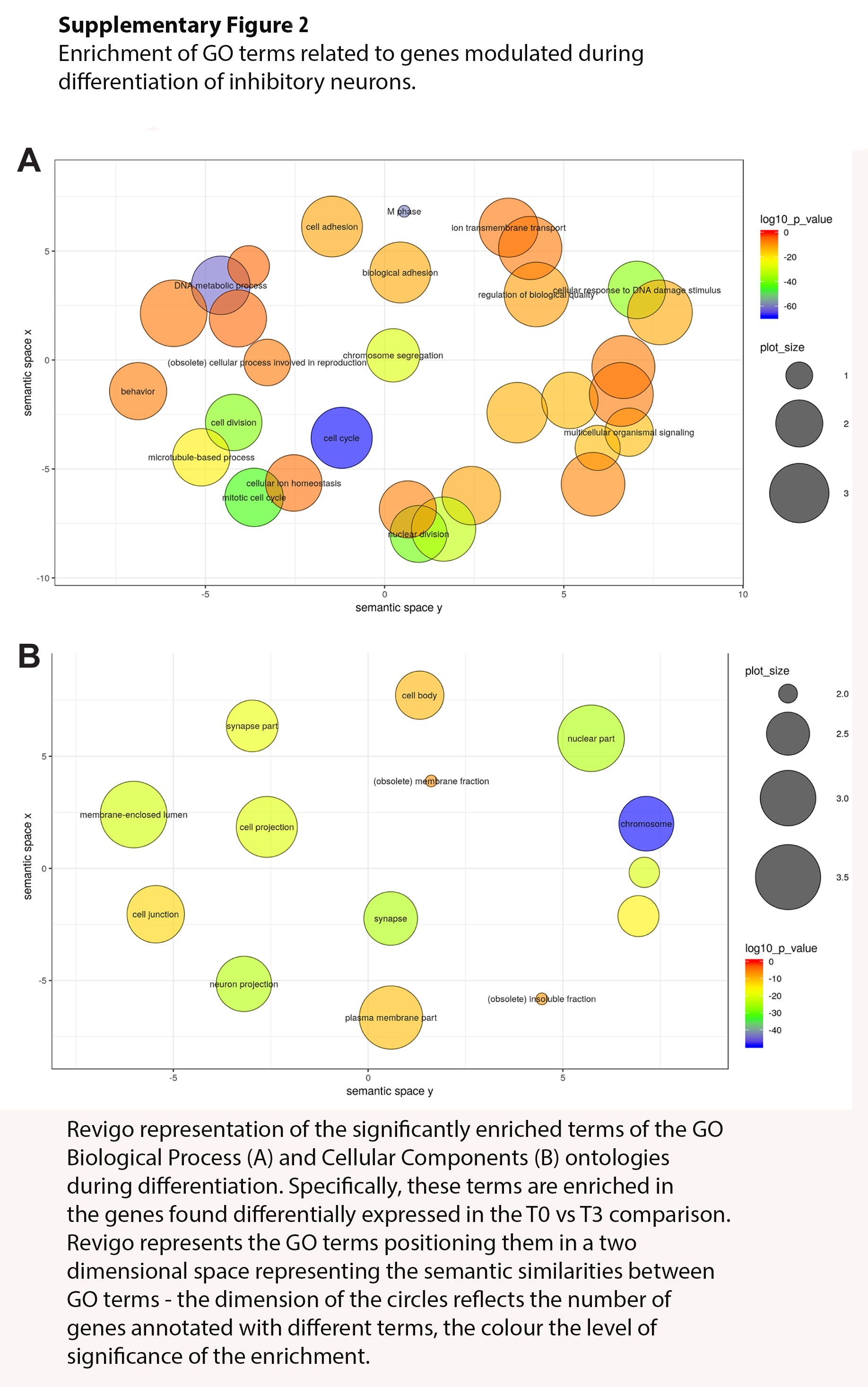

Supplement: Supplementary file 6 [file Image_2.TIF]

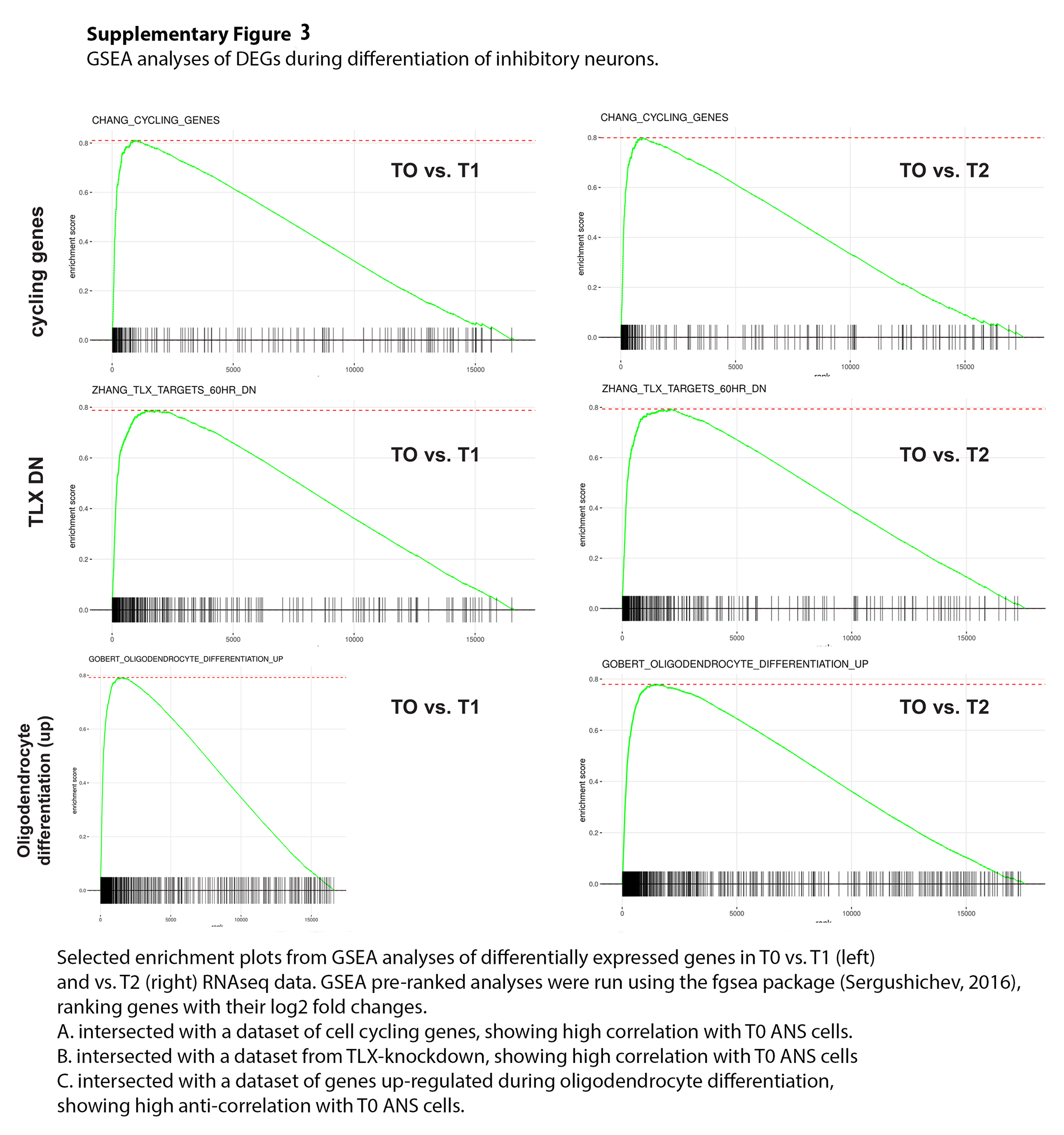

Supplement: Supplementary file 7 [file Image_3.tif]
